# Supplementary material for: Unpacking the multilingualism continuum: An investigation of language variety co-activation in simultaneous interpreters
Source: PLoS One. 2023 Nov 28;18(11):e0289484. doi: 10.1371/journal.pone.0289484 (PMC10684095; doi:10.1371/journal.pone.0289484)
Supplement: S1 Appendix — (PDF) [file pone.0289484.s001.pdf]

Condition 1: Intervariety competition

Target	EN	FR	Frequency class	Syllables	Phonemes	L1' competitor	EN	FR	Frequency class	Syllables	Phonemes	Target-competitor phoneme overlap	Filler 1	EN	FR	Frequency class	Syllables	Phonemes	Filler 2	EN	FR	Frequency class	Syllables	Phonemes	Average Frequency class	SD FC	Average syllable count	SD syllable count	Average phoneme count	SD phoneme count
Birne	*pear*	*poire*	14	2	5	Bibeli - Kücken	*chick*	*poussin*	18	3	6	2	Hocker	*stool*	*tabouret*	14	2	5	Steinschleuder	*slingshot*	*lance-pierre*	17	3	10	15.75	2.06	2.5	0.6	6.5	2.4
Brücke	*bridge*	*pont*	10	2	5	Brünneli - Waschbecken	*sink*	*lavabo*	15	3	7	3	Kette	*chain*	*chaîne*	12	2	4	Rutschbahn	*slide*	*toboggan*	16	2	6	13.25	2.75	2.25	0.5	5.5	1.3
Finger	*finger*	*doigt*	10	2	5	Finken - Hausschuhe	*slippers*	*pantoufles*	17	2	6	3	Einhorn	*unicorn*	*licorne*	15	2	5	Bumerang	*boomerang*	*boomerang*	15	3	8	14.25	2.99	2.25	0.5	6	1.4
Geige	*violine*	*violine*	13	2	4	Geiss - Ziege	*goat*	*chèvre*	13	1	3	2	Erdbeere	*strawberry*	*fraise*	13	3	7	Bombe	*bomb*	*bombe*	11	2	5	12.5	1.00	2	0.8	4.75	1.7
Gurke	*cucumber*	*concombre*	15	2	5	Gufä - Stecknadel	*pin*	*épingle*	16	2	4	2	Ei	*egg*	*oeuf*	12	1	1	Berg	*mountain*	*montagne*	10	1	4	13.25	2.75	1.5	0.6	3.5	1.7
Gürtel	*belt*	*ceinture*	12	2	5	Güggel - Hahn	*shelf*	*étagère*	11	2	5	2	Luftballon	*balloon*	*ballon*	15	3	9	Löffel	*spoon*	*cuillère*	13	2	5	12.75	1.71	2.25	0.5	6	2.0
Haar	*hair*	*cheveux*	11	1	3	Haag - Zaun	*fence*	*clôture*	12	1	3	2	Banane	*banana*	*banane*	14	3	6	Massstab	*ruler*	*règle*	14	2	6	12.75	1.50	1.75	1.0	4.5	1.7
Herd	*stove, cooker*	*cuisinière*	12	1	4	Herdöpfel - Kartoffel	*potato*	*pomme de terre*	13	3	8	4	Bär	*bear*	*ours*	11	1	3	Mond	*moon*	*lune*	11	1	4	11.75	0.96	1.5	1.0	4.75	2.2
Lüster	*chandelier*	*lustre*	18	2	6	Lüüti - Klingel, Türklingel	*doorbell*	*sonnette*	16	2	4	2	Pyramide	*pyramide*	*pyramide*	14	4	8	Delfin	*dolphin*	*dauphin*	15	2	6	15.75	1.71	2.5	1.0	6	1.6
Nagel	*nail*	*clou*	12	2	5	Natel - Handy	*mobile/cell phone*	*portable*	9	2	5	2	Kaktus	*cactus*	*cactus*	16	2	6	Feuerzeug	*lighter*	*briquet*	15	3	7	13	3.16	2.25	0.5	5.75	1.0
Nashorn	*rhino*	*rhinocéros*	16	2	7	Nastüechli - Taschentuch	*tissue*	*mouchoir*	15	3	8	3	Badewanne	*bathtub*	*beignoire*	13	4	8	Hubschrauber	*chopper, helicopter*	*helicoptère*	11	3	9	13.75	2.22	3	0.8	8	0.8
Nuss	*nut*	*noix*	14	1	3	Nuggi - Schnuller	*pacifier*	*lollette/tetine*	15	2	4	2	Löwe	*lion*	*lion*	13	2	4	Handschuh	*glove*	*gant*	15	2	6	14.25	0.96	1.75	0.5	4.25	1.3
Rüstung	*armor*	*armure*	14	2	6	Rüebli - Karotte	*carrot*	*carotte*	14	2	5	2	Schildkröte	*turtle*	*tortue*	15	3	9	Waage	*scale*	*balance*	12	2	4	13.75	1.26	2.25	0.5	6	2.2
Sakko	*dress jacket*	*veste*	15	2	4	Sackmesser - Taschenmesser	*pocket knife*	*couteau de poche*	16	3	8	3	Lama	*llama*	*lama*	12	2	4	Maske	*mask*	*masque*	12	2	5	13.75	2.06	2.25	0.5	5.25	1.9
Schnorchel	*snorkel*	*tuba*	17	2	7	Schnoogg - Mücke (Moskito)	*mosquito*	*moustique*	13	1	4	3	Ordner	*folder, binder*	*classeur*	13	2	6	Hammer	*hammer*	*marteau*	11	2	5	13.5	2.52	1.75	0.5	5.5	1.3
Schokolade	*chocolate*	*chocolat*	11	4	8	Schoppä - Fläschchen	*baby bottle,*	*biberon*	15	2	4	2	Trommel	*drum*	*tambour*	14	2	6	Zwiebel	*onion*	*oignion*	13	2	6	13.25	1.71	2.5	1.0	6	1.6
Stern	*star*	*étoile*	10	1	5	Stägä - Treppe	*stairs (scht vs st)*	*escalier*	12	2	5	3	Bank	*bench*	*banc*	8	1	4	Huhn	*chicken*	*poule*	13	1	3	10.75	2.22	1.25	0.5	4.25	1.0
Storch	*stork*	*cigogne*	14	1	5	Storä - Jalousien	*blinds*	*volets*	16	2	5	4	Daumen	*thumb*	*pouce*	12	2	5	Teller	*plate*	*assiette*	12	2	5	13.5	1.91	1.75	0.5	5	0.0
Sträfling	*convict*	*prisonnier*	18	2	8	Strähl - Haarbürste	*hairbrush*	*brosse à cheveux*	19	1	5	4	Mikrofon	*microphone*	*microphone*	12	3	8	Affe	*monkey*	*singe*	14	2	3	15.75	3.30	2	0.8	6	2.4
Stuhl	*chair*	*chaise*	11	1	4	Stumpä - Zigarre	*cigar*	*cigare*	15	2	6	3	Ente	*duck*	*canard*	13	2	4	Nadel	*needle*	*aguille*	13	2	5	13	1.63	1.75	0.5	4.75	1.0
Tee	*tea*	*thé*	11	1	2	Thek - Ranzen, Schultasche	*schoolbag*	*cartable*	15	1	3	2	Hai	*shark*	*requin*	13	1	3	Spritze	*syringe*	*seringue*	14	2	6	13.25	1.71	1.25	0.5	3.5	1.7
Törtchen	*tartlet*	*tartelette*	17	2	7	Töff - Motorrad	*motorbike*	*moto*	11	1	3	2	Gabel	*fork*	*fourchette*	14	2	5	Biene	*bee*	*abeille*	14	2	4	14	2.45	1.75	0.5	4.75	1.7
Trichter	*funnel*	*entonnoir*	15	2	7	Tritt - Stufe	*step*	*marche*	11	1	4	3	Flügel (Vogel)	*wing*	*aile*	11	2	6	Pistole	*pistol, gun*	*pistolet*	12	3	7	12.25	1.89	2	0.8	6	1.4
Zelt	*tent*	*tente*	11	1	4	Zältli - Bonbon	*candy*	*bonbon*	15	2	6	4	Auge	*eye*	*oeuil*	9	2	3	Salat	*salad*	*salade*	12	2	5	11.75	2.50	1.75	0.5	4.5	1.3
Zündschlüssel	*ignition key*	*clé de contact*	16	3	10	Zündhölzli - Streichholz	*match*	*allumette*	16	3	10	4	Schuh	*shoe*	*chaussure*	12	1	2	Blume	*flower*	*fleur*	13	2	5	14.25	2.06	2.25	1.0	6.75	3.9
Averages			13.5	1.8	5.4				14.3	2.0	5.2	2.7				12.8	2.2	5.2				13.12	2.08	5.56						
SDs			2.6	0.7	1.8				2.4	0.7	1.8	0.8				1.8	0.9	2.1				1.8	0.6	1.7						

Condition 2: Intravariety competition

Target	EN	FR				L1 competitor	EN	FR				Target-competitor phoneme overlap	Filler 1	EN	FR				Filler 2	EN	FR									
Balkon	*balcony*	*balcon*	11	2	6	Ball	*ball*	*ballon*	9	1	3	3	Feuer	*fire*	*feu*	9	2	4	Zebra	*zebra*	*zèbre*	15	2	5	11	2.83	1.75	0.5	4.5	1.3
Batterie	*battery*	*batterie*	13	3	6	Bagger	*excavator, digger*	*excavateur*	12	2	5	2	Tanne	*fir tree*	*sapin*	14	2	4	Kürbis	*pumpkin*	*courge*	14	2	6	13.25	0.96	2.25	0.5	5.25	1.0
Beil	*axe*	*hache*	15	1	3	Bein	*leg*	*jambe*	11	1	3	2	Känguru	*kangaroo*	*kangourou*	15	3	7	Ananas	*pineapple*	*ananas*	14	3	6	13.75	1.89	2	1.2	4.75	2.1
Brief	*letter*	*lettre*	10	1	4	Brille	*glasses*	*lunettes*	12	2	5	3	Kerze	*candle*	*bougie*	13	2	5	Skorpion	*scorpion*	*scorpion*	15	3	8	12.5	2.08	2	0.8	5.5	1.7
Bus	*bus*	*bus*	10	1	3	Buch	*book*	*livre*	8	1	3	2	Absatz	*heel*	*talon*	11	2	5	Feder	*feather*	*plume*	12	2	5	10.25	1.71	1.5	0.6	4	1.2
Gespenst	*ghost*	*fantôme*	14	2	8	Geschenk	*gift*	*cadeau*	10	2	6	3	Pelikan	*pelican*	*pélican*	15	3	7	Träne	*tear*	*larme*	14	2	5	13.25	2.22	2.25	0.5	6.5	1.3
Glocke	*bell*	*cloche*	12	2	5	Globus	*globe*	*globe*	12	2	6	3	Schwan	*swan*	*cigne*	13	1	4	Rose	*rose*	*rose*	11	2	4	12	0.82	1.75	0.5	4.75	1.0
Haken	*hook*	*crochet*	12	2	5	Hantel	*dumbbell, barbell*	*haltère*	17	2	6	2	Kissen	*pillow*	*coussin*	13	2	5	Lupe	*magnifying glass*	*loupe*	11	2	4	13.25	2.63	2	0.0	5	0.8
Huf	*hoof*	*sabot*	17	1	3	Hut	*hat*	*chapeau*	10	1	3	2	Fenster	*window*	*fenêtre*	9	2	7	Käse	*cheese*	*fromage*	11	2	4	11.75	3.59	1.5	0.6	4.25	1.9
Kamel	*camel*	*chameau*	14	2	5	Kamm	*comb*	*peigne*	14	1	3	3	Lichtschalter	*light switch*	*interrupteur*	16	3	10	Stein	*stone*	*pierre*	10	1	4	13.5	2.52	1.75	1.0	5.5	3.1
Kanone	*canon*	*cannon*	15	3	6	Kanu	*canoo*	*canoë*	15	2	4	3	Sanduhr	*hourglass, sandglass*	*sablier*	17	2	6	Telefon	*phone, telephone*	*téléphone*	10	3	7	14.25	2.99	2.5	0.6	5.75	1.3
Knochen	*bone*	*os*	11	2	6	Knopf	*button*	*bouton*	13	1	4	3	Spinne	*spider*	*araignée*	14	2	5	Apfel	*apple*	*pomme*	12	2	4	12.5	1.29	1.75	0.5	4.75	1.0
Krokodil	*crocodile*	*crocodile*	14	3	8	Krone	*crown*	*couronne*	11	2	5	3	Rasenmäher	*lawn mower*	*tondeuse*	15	4	10	Hose	*pants, trousers*	*pantalons*	11	2	4	12.75	2.06	2.75	1.0	6.75	2.8
Leiter	*ladder*	*échelle*	8	2	5	Leinwand	*screen, canvas*	*écran, toile*	11	2	7	2	Vase	*vase*	*vase*	15	2	4	Anker	*anchor*	*ancre*	13	2	4	11.75	2.99	2	0.0	5	1.4
Papier	*paper*	*papier*	10	2	5	Papagei	*parrot*	*perroquet*	15	3	6	3	Reissverschluss	*zipper*	*zip*	18	3	10	Schneemann	*snowman*	*bon homme de neige*	14	2	6	14.25	3.30	2.5	0.6	6.75	2.2
Pfeil	*arrow*	*flèche*	13	1	3	Pfeife	*pipe*	*pipe*	14	2	4	2	Kühlschrank	*fridge*	*frigo*	12	2	8	Ventilator	*fan*	*ventilator*	16	4	10	13.75	1.71	2.25	1.3	6.25	3.3
Pillen	*pills*	*pillules*	14	2	5	Pilz	*mushroom*	*champignon*	13	1	4	3	Krebs	*crab*	*crabe*	11	1	5	Tasche	*bag, purse*	*sac*	10	2	4	12	1.83	1.5	0.6	4.5	0.6
Pinguin	*penguin*	*pinguin*	15	3	7	Pinsel	*paint brush*	*pinceau*	13	2	6	2	Sofa	*couch, sofa*	*sofa*	12	2	4	Baum	*tree*	*arbre*	10	1	3	12.5	2.08	2	0.8	5	1.8
Schale	*bowl*	*bol*	12	2	4	Schal	*shawl*	*écharpe*	13	1	3	3	Esel	*donkey, ass*	*âne*	12	2	4	Palme	*palm tree*	*palmier*	13	2	5	12.5	0.58	1.75	0.5	4	0.8
Schaukel	*swing*	*balançoire*	15	2	5	Schaufel	*shovel*	*pelle*	14	2	5	2	Harfe	*harp*	*harpe*	14	2	5	Katze	*cat*	*chat*	11	2	4	13.5	1.73	2	0.0	4.75	0.5
Schwert	*sword*	*épé*	13	1	5	Schwein	*pig*	*cochon*	12	1	4	2	Pullover	*sweater*	*pull*	13	3	7	Blatt (>Baum)	*leaf*	*feuille*	10	1	4	12	1.41	1.5	1.0	5	1.4
Sonne	*sun*	*soleil*	9	2	4	Socke	*sock*	*chaussette*	13	2	4	2	Koffer	*suitcase*	*valise*	11	2	5	Hund	*dog*	*chien*	9	1	4	10.5	1.91	1.75	0.5	4.25	0.5
Tisch	*table*	*table*	9	1	3	Tiger	*tiger*	*tigre*	11	2	5	2	Rucksack	*bagpack*	*sac à dos*	12	2	6	Pferd	*horse*	*cheval*	11	1	4	10.75	1.26	1.5	0.6	4.5	1.3
Vulkan	*vulcano*	*volcan*	12	2	6	Wurm	*worm*	*verre*	13	1	4	2	Hand	*hand*	*main*	8	1	4	Leuchtturm	*light house*	*phare*	14	2	8	11.75	2.63	1.5	0.6	5.5	1.9
Wolle	*wool*	*laine*	13	2	4	Wolke	*cloud*	*nuage*	12	2	5	3	Amboss	*anvil*	*enclume*	16	2	5	Dinosaurier	*dinosaur*	*dinosaure*	13	5	10	13.5	1.73	2.75	1.5	6	2.7
Averages			12.4	1.9	5.0				12.3	1.6	4.5	2.5				13.1	2.2	5.8				12.2	2.1	5.3						
SDs			2.3	0.7	1.5				2.0	0.6	1.2	0.5				2.5	0.7	2.0				2.0	0.9	1.9						

Condition 3: no competition

Target	EN	FR				Filler 1	EN	FR				Target-competitor phoneme overlap	Filler 2	EN	FR				Filler 3	EN	FR									
Ampel	*traffic light*	*feux*	12	2	5	Blitz	*lightening*	*éclair*	13	1	4	0	Eule	*owl*	*chouette*	15	2	3	Kleiderbügel	*clotheshanger*	*cintre*	17	4	11	14.25	2.22	2.25	1.3	5.75	3.6
Bart	*beard*	*barbe*	12	1	4	Schaf	*sheep*	*mouton*	13	1	3	0	Fernseher	*TV*	*télé*	11	3	9	Kuchen	*cake*	*gâteau*	10	2	5	11.5	1.29	1.75	1.0	5.25	2.6
Besen	*broom*	*balai*	13	2	5	Aschenbecher	*ashtray*	*cendrier*	15	4	8	0	Wasserhahn	*tap, faucet*	*robinet*	15	3	8	Krug	*jug, jar*	*pichet*	13	1	4	14	1.15	2.5	1.3	6.25	2.1
Bleistift	*pencil*	*crayon*	14	2	8	Schlange	*snake*	*serpent*	12	2	5	0	Mais	*corn*	*mais*	12	1	3	Weinglas	*wine glass*	*verre à vin*	17	2	7	13.75	2.36	1.75	0.5	5.75	2.2
Elefant	*elefant*	*éléphant*	13	3	7	Zigarette	*cigarette*	*cigarette*	11	4	8	0	Bett	*bed*	*lit*	10	1	3	Münze	*coin*	*pièce de monnaie*	13	2	5	11.75	1.50	2.5	1.3	5.75	2.2
Flasche	*bottle*	*bouteille*	11	2	5	Nase	*nose*	*nez*	10	2	4	0	Pfau	*peacock*	*paon*	15	1	2	Schere	*scissors*	*ciseaux*	13	2	4	12.25	2.22	1.75	0.5	3.75	1.3
Fuchs	*fox*	*renard*	11	1	4	Schraubenzieher	*screwdriver*	*tournevis*	16	4	11	0	Wassermelone	*watermelon*	*pastèque*	17	5	11	Regenbogen	*rainbow*	*arc-en-ciel*	14	4	10	14.5	2.65	3.5	1.7	9	3.4
Kleid	*dress*	*robe*	12	1	4	Ameise	*ant*	*fourmie*	14	3	5	0	Saxofon	*saxophone*	*saxophone*	15	3	7	Burg	*castle*	*château fort*	10	1	4	12.75	2.22	2	1.2	5	1.4
Kugelschreiber	*pen*	*stylo à bille*	15	4	11	Muschel	*sea shell*	*coquillage*	14	2	5	0	Fahne	*flag*	*drapeau*	12	2	4	Biber	*biever*	*castor*	14	2	5	13.75	1.26	2.5	1.0	6.25	3.2
Panda	*panda*	*panda*	14	2	5	Ast	*branch*	*branche*	13	1	3	0	Tasse	*cup*	*tasse*	12	2	4	Hemd	*shirt*	*chemise*	12	1	4	12.75	0.96	1.5	0.6	4	0.8
Perücke	*wig*	*perruque*	14	3	6	Messer	*knife*	*couteau*	10	2	5	0	Lampe	*lamp*	*lampe*	13	2	5	Robbe	*seal*	*phoque*	14	2	4	12.75	1.89	2.25	0.5	5	0.8
Pirat	*pirat*	*pirate*	15	2	5	Schlüssel	*key*	*clé*	11	2	6	0	Teleskop	*telescope*	*téléscope*	14	3	8	Brot	*bread*	*pain*	10	1	4	12.5	2.38	2	0.8	5.75	1.7
Radio	*radio*	*radio*	9	3	5	Bratpfanne	*frying pan*	*poêle*	16	3	8	0	Säge	*saw*	*scie*	14	2	4	Dusche	*shower*	*douche*	12	2	4	12.75	2.99	2.5	0.6	5.25	1.9
Schachtel	*box*	*boîte*	14	2	6	Korkenzieher	*corkscrew*	*tire-bouchon*	17	4	11	0	Tintenfisch	*octopus*	*octopus*	15	3	9	Haus	*house*	*maison*	7	1	3	13.25	4.35	2.5	1.3	7.25	3.5
Schmetterling	*butterfly*	*papillon*	13	3	9	Krawatte	*tie*	*cravatte*	13	3	7	0	Büroklammer	*paper-clip*	*trombone*	18	4	10	Glühbirne	*light bulb*	*ampoule*	14	3	8	14.5	2.38	3.25	0.5	8.5	1.3
Schnecke	*snail*	*escargot*	15	2	5	Fuss	*foot*	*pied*	12	1	3	0	Grill	*bbq*	*bbq, grill*	12	1	4	Nest	*nest*	*nid*	13	1	4	13	1.41	1.25	0.5	4	0.8

Skelett	*skeleton*	*squelette*	14	2	6	Pflanze	*plant*	*plante*	12	2	6	0	Trauben	*grapes*	*raisin*	13	2	6	Kassette	*tape*	*cassette*	15	3	6	13.5	1.29	2.25	0.5	6	0.0
Ski	*skis*	*skis*	11	1	2	Ohr	*ear*	*oreille*	11	1	2	0	Zitrone	*lemon*	*citron*	14	3	7	Kamera	*camera*	*caméra*	9	3	6	11.25	2.06	2	1.2	4.25	2.6
Spiegel	*mirror*	*glace*	10	2	6	Iglu	*igloo*	*iglou*	16	2	4	0	Fledermaus	*bat*	*chauvesouris*	15	3	9	Tomate	*tomato*	*tomate*	15	3	6	14	2.71	2.5	0.6	6.25	2.1
Stecker	*plug*	*prise*	14	2	6	Gans	*goose*	*oie*	13	1	4	0	Akkordeon	*accordion*	*accordéon*	13	4	8	Rock	*skirt*	*jupe*	10	1	3	12.5	1.73	2	1.4	5.25	2.2
Teppich	*rug*	*tapis*	11	2	5	Fisch	*fish*	*poisson*	11	1	3	0	Spitzer	*pencil sharpener*	*taille-crayon*	15	2	6	Arm	*arm*	*bras*	10	1	3	11.75	2.22	1.5	0.6	4.25	1.5
Uhr	*clock*	*horloge*	5	1	2	Drachen	*kite*	*cerf-volant*	13	2	6	0	Fass	*barrel, cask*	*tonneau*	12	1	3	Kuh	*cow*	*vache*	12	1	2	10.5	3.70	1.25	0.5	3.25	1.9
Vogel	*bird*	*oiseau*	11	2	5	Trompete	*trumpet*	*trompette*	13	13	8	0	Schraube	*screw*	*vice*	14	2	5	Pflaster	*band-aid, plaster*	*pansement*	13	2	7	12.75	1.26	4.75	5.5	6.25	1.5
Vorhang	*curtain*	*rideau*	12	2	6	Schiff	*ship*	*bateau*	10	1	3	0	Korb	*basket*	*corbeille*	12	1	4	Fliege	*fly*	*mouche*	14	2	5	12	1.63	1.5	0.6	4.5	1.3
Wäscheklammer	*clothespin*	*pince à linge*	19	4	10	Jacke	*jacket*	*veste*	12	2	4	0	Kirche	*church*	*église*	7	2	5	Seepferdchen	*sea horse*	*hippocampe*	16	3	9	13.5	5.20	2.75	1.0	7	2.9
Averages			12.6	2.1	5.7				12.8	2.6	5.4	0.0				13.4	2.3	5.9				12.7	2.0	5.3						
SDs			2.6	0.8	2.1				2.0	2.4	2.5	0.0				2.3	1.1	2.6				2.5	1.0	2.3						

Practice trials

Target	EN	FR				L1 competitor	EN	FR				Target-competitor phoneme overlap	Filler 1	EN	FR				Filler 2	EN	FR									
Bohrmaschine	*drill*	*perceuse*	16	4	9	Hängematte	*hammock*	*hamac*	15	4	8	0	Frosch	*frog*	*grenouille*	14	1	4	Schrank	*closet*	*armoire*	12	1	5	14.25	1.71	2.5	1.7	6.5	2.4
Gitarre	*guitar*	*guitarre*	11	3	6	Giraffe	*giraffe*	*girafe*	15	3	6	2	Zahn	*tooth*	*dent*	12	1	3	Mütze	*hat*	*bonnet*	13	2	4	12	1.71	2.25	1.0	4.75	1.5
Bild	*painting*	*tableau*	7	1	4	Billet - Ticket, Fahrschein	*ticket*	*billet*	12	2	5	3	Schirm	*umbrella*	*parapluie*	13	1	4	Diamant	*diamond*	*diamant*	15	3	7	11.75	3.40	1.75	1.0	5	1.4
Averages			11.3	2.7	6.3				14.0	3.0	6.3	1.7				13.0	1.0	3.7				13.3	2.0	5.3						
SDs			3.6	1.0	2.3				3.0	2.4	2.4	0.7				3.1	1.1	2.4				3.0	0.9	2.0						
