# Supplementary material for: Unpacking the multilingualism continuum: An investigation of language variety co-activation in simultaneous interpreters
Source: PLoS One. 2023 Nov 28;18(11):e0289484. doi: 10.1371/journal.pone.0289484 (PMC10684095; doi:10.1371/journal.pone.0289484)
Supplement: S2 Appendix — (PDF) [file pone.0289484.s002.pdf]

Production Task: Sentence List 1

Sentences Condition 1	Targets EN	Frequency (source: BCN)	Syllable count	Phoneme count	Combined frequency indications of actor, verb & target	Combined syllable counts	Combined phoneme counts
The queen remembered the [target].	*pear*	*222*	*1*	*3*	*9376*	*5*	*16*
The painter sketched the [target].	*bridge*	*6292*	*1*	*4*	*15657*	*5*	*14*
The boy gazed at the [target].	*finger*	*3013*	*2*	*5*	*1793*	*7*	*22*
The child stared at the [target].	*violin*	*558*	*2*	*6*	*24347*	*5*	*17*
The secretary asked about the the [target].	*cucumber*	*205*	*3*	*8*	*839*	*8*	*23*
The artist painted the [target].	*belt*	*2045*	*1*	*4*	*26727*	*6*	*16*
The reporter talked about the [target].	*hair*	*13455*	*1*	*3*	*18923*	*6*	*19*
The father thought about the [target].	*stove*	*580*	*1*	*4*	*3725*	*5*	*13*
The girl mentioned the [target].	*chandelier*	*83*	*3*	*8*	*7141*	*10*	*26*
The guest admired the [target].	*nail*	*723*	*1*	*3*	*28756*	*4*	*12*
The conductor stood next to the [target].	*rhino*	*219*	*2*	*4*	*21145*	*7*	*15*
The man pointed at the [target]	*nut*	*627*	*1*	*3*	*59401*	*5*	*14*
The mother saw the [target].	*armour*	*856*	*2*	*5*	*137929*	*5*	*12*
The president drew the [target].	*dress jacket*	*2771*	*2*	*5*	*8235*	*4*	*15*
The chairperson observed the [target].	*snorkel*	*19*	*2*	*6*	*25428*	*6*	*17*
The scientist moved towards the [target].	*chocolate*	*1931*	*3*	*6*	*26065*	*5*	*16*
The investigator forgot about the [target].	*star*	*6330*	*1*	*4*	*18520*	*7*	*19*
The researcher dreamed about the [target].	*stork*	*90*	*1*	*5*	*5359*	*6*	*18*
The witness described the [target].	*convict*	*244*	*2*	*7*	*4451*	*9*	*24*
The student presented the [target].	*chair*	*7231*	*1*	*3*	*15517*	*10*	*25*
The lady indicated the [target].	*tea*	*7942*	*1*	*2*	*39411*	*6*	*19*
The teacher hid the [target].	*tartlet*	*42*	*2*	*7*	*10969*	*5*	*15*
The king held the [target].	*funnel*	*161*	*2*	*5*	*5711*	*7*	*21*
The visitor photographed the [target].	*tent*	*1090*	*1*	*4*	*6831*	*6*	*21*
The woman looked at the [target].	*ignition*	*256*	*3*	*7*	*36693*	*7*	*18*
				AVG	22358	6	18
				SD	27781	2	4

Sentences Condition 2	Targets EN	Frequency	Syllables	Phonemes	Combined frequency indications of actor, verb & target	Combined syllable counts	Combined phoneme counts
The woman admired the [target].	*balcony*	*912*	*3*	*7*	*6319*	*6*	*21*
The visitor saw the [target].	*battery*	*1265*	*3*	*6*	*35904*	*5*	*15*
The king gazed at the [target].	*axe*	*738*	*1*	*2*	*4122*	*6*	*19*
The child held the [target].	*letter*	*12993*	*2*	*5*	*49480*	*4*	*13*
The teacher observed the the [target].	*bus*	*5225*	*1*	*3*	*42032*	*8*	*23*
The lady drew the [target].	*ghost*	*1300*	*1*	*4*	*23242*	*7*	*20*
The student forgot about the [target].	*bell*	*3239*	*1*	*3*	*15675*	*3*	*13*
The witness stared at the [target].	*hook*	*1380*	*1*	*3*	*3872*	*6*	*15*
The researcher stood next to the [target].	*hoof*	*124*	*1*	*3*	*4728*	*11*	*26*
The investigator indicated the [target].	*camel*	*358*	*2*	*5*	*16416*	*4*	*11*
The scientist looked at the [target].	*cannon*	*628*	*2*	*5*	*17605*	*4*	*11*
The chairperson mentioned the [target]	*bone*	*2348*	*1*	*3*	*77678*	*6*	*17*
The president thought about the [target].	*crocodile*	*247*	*3*	*8*	*37671*	*6*	*17*
The man remembered the [target].	*ladder*	*1261*	*2*	*5*	*5501*	*8*	*21*
The conductor presented the [target].	*paper*	*16755*	*2*	*5*	*19453*	*9*	*24*
The guest pointed at the [target].	*arrow*	*1007*	*2*	*3*	*12741*	*4*	*13*
The girl sketched the [target].	*pills*	*544*	*1*	*4*	*5823*	*5*	*14*
The father asked about the [target].	*penguin*	*467*	*2*	*7*	*1088*	*7*	*21*
The reporter hid the [target].	*bowl*	*2335*	*1*	*3*	*17276*	*7*	*18*
The artist moved towards the [target].	*swing*	*1880*	*1*	*4*	*20783*	*8*	*23*
The secretary dreamed about the [target].	*sword*	*1330*	*1*	*4*	*11178*	*7*	*20*

The mother photographed the [target].	sun	11025	1	3		15424	5	15
The boy described the [target].	table	19128	2	4		116313	7	16
The painter talked about the [target].	volcano	378	3	7		4054	5	16
The queen painted the [target].	wool	1737	1	3		23446	8	18
					AVG	23513	6	18
					SD	26132	2	4

Sentences Condition 3	Targets EN	Frequency	Syllables	Phonemes		Combined frequency indications of actor, verb & target	Combined syllable counts	Combined phoneme counts
The scientist mentioned the [target].	traffic light	37	3	9		4973	9	24
The witness admired the [target].	beard	882	1	4		21529	5	14
The lady held the [target].	broom	323	1	4		3846	6	19
The king hid the [target].	pencil	1132	2	6		34218	4	12
The painter indicated the [target].	elephant	892	3	7		2405	7	21
The secretary saw the [target].	bottle	3900	2	4		24837	5	14
The father gazed at the [target].	fox	2245	1	3		14684	5	18
The researcher moved towards the [target].	dress	4739	1	4		3212	5	15
The chairperson photographed the [target].	pen	2072	1	3		7844	9	23
The conductor observed the [target].	panda	175	2	5		19077	4	12
The teacher remembered the [target].	wig	309	1	3		22539	4	12
The visitor presented the [target]	pirate	267	2	5		59543	5	16
The queen talked about the [target].	radio	8454	3	5		28187	6	19
The boy pointed at the [target].	box	8363	1	3		6075	9	22
The artist forgot about the [target].	butterfly	623	3	8		31539	7	21
The girl stood next to the [target].	snail	288	1	4		19976	7	17
The man sketched the [target].	skeleton	627	3	8		6229	6	20
The student dreamed about the [target].	skis	215	1	3		6208	7	23
The child drew the [target].	mirror	3642	2	5		6496	8	23
The reporter described the [target].	plug	876	1	4		131895	7	15
The guest asked about the [target].	rug	686	1	3		12133	6	20
The woman stared at the [target].	clock	2764	1	4		27134	6	17
The investigator thought about the [target].	bird	3823	1	4		27035	8	22
The president looked at the [target].	curtain	1326	2	6		3996	6	16
The mother painted the [target].	clothespin	654	2	8		24858	5	16
					AVG	22019	6	18
					SD	26513	2	4

Sentences Practice trials	Targets EN	Frequency	Syllables	Phonemes		Combined frequency indications of actor, verb & target	Combined syllable counts	Combined phoneme counts
The astronaut ran away from the [target].	drill	1033	1	3		1256	8	22
The clown sat next to the [target].	guitar	2401	2	5		2817	6	18
The baby contemplated the [target].	painting	4161	2	6		13220	8	22
					AVG	5764	7	21
					SD	6504	1	2

Production Task: Sentence List 2

Sentences Condition 1	Targets EN	Frequency (source: BCN)	Syllable count	Phoneme count		Combined frequency indications of actor, verb & target	Combined syllable counts	Combined phoneme counts
The painter sketched the [target].	*pear*	*222*	*1*	*3*		*2165*	*4*	*14*
The boy gazed at the [target].	*bridge*	*6292*	*1*	*4*		*18936*	*4*	*13*
The child stared at the [target].	*finger*	*3013*	*2*	*5*		*26802*	*5*	*16*
The secretary asked about the the [target].	*violin*	*558*	*2*	*6*		*15870*	*9*	*23*
The artist painted the [target].	*cucumber*	*205*	*3*	*8*		*7536*	*7*	*20*
The reporter talked about the [target].	*belt*	*2045*	*1*	*4*		*7110*	*7*	*20*
The father thought about the [target].	*hair*	*13455*	*1*	*3*		*39602*	*6*	*15*
The girl mentioned the [target].	*stove*	*580*	*1*	*4*		*19420*	*4*	*15*
The guest admired the [target].	*chandelier*	*83*	*3*	*8*		*3085*	*7*	*18*
The conductor stood next to the [target].	*nail*	*723*	*1*	*3*		*1343*	*7*	*22*
The man pointed at the [target]	*rhino*	*219*	*2*	*4*		*58993*	*6*	*15*
The mother saw the [target].	*nut*	*627*	*1*	*3*		*137700*	*4*	*10*
The president drew the [target].	*armour*	*856*	*2*	*5*		*23513*	*6*	*17*
The chairperson observed the [target].	*dress jacket*	*2771*	*2*	*5*		*4545*	*7*	*21*
The scientist moved towards the [target].	*snorkel*	*19*	*2*	*6*		*2539*	*8*	*24*
The investigator forgot about the [target].	*chocolate*	*1931*	*3*	*6*		*2742*	*12*	*28*
The researcher dreamed about the [target].	*star*	*6330*	*1*	*4*		*11689*	*7*	*22*
The witness described the [target].	*stork*	*90*	*1*	*5*		*6677*	*5*	*19*
The student presented the [target].	*convict*	*244*	*2*	*7*		*32424*	*7*	*23*
The lady indicated the [target].	*chair*	*7231*	*1*	*3*		*20434*	*7*	*16*
The teacher hid the [target].	*tea*	*7942*	*1*	*2*		*18869*	*4*	*10*
The king held the [target].	*tartlet*	*42*	*2*	*7*		*28637*	*4*	*14*
The visitor photographed the [target].	*funnel*	*161*	*2*	*5*		*4782*	*8*	*22*
The woman looked at the [target].	*tent*	*1090*	*1*	*4*		*37527*	*5*	*15*
The queen remembered the [target].	*ignition*	*256*	*3*	*7*		*26099*	*7*	*20*
					AVG	22362	6	18
					SD	28036	2	4

Sentences Condition 2	Targets EN	Frequency	Syllables	Phonemes		Combined frequency indications of actor, verb & target	Combined syllable counts	Combined phoneme counts
The visitor saw the [target].	*balcony*	*912*	*3*	*7*		*115960*	*7*	*17*
The king gazed at the [target].	*battery*	*1265*	*3*	*6*		*16943*	*6*	*15*
The child held the [target].	*axe*	*738*	*1*	*2*		*37225*	*3*	*10*
The teacher observed the the [target].	*letter*	*12993*	*2*	*5*		*23192*	*6*	*17*
The lady drew the [target].	*bus*	*5225*	*1*	*3*		*21530*	*4*	*10*
The student forgot about the [target].	*ghost*	*1300*	*1*	*4*		*9239*	*7*	*21*
The witness stared at the [target].	*bell*	*3239*	*1*	*3*		*5913*	*5*	*16*
The researcher stood next to the [target].	*hook*	*1380*	*1*	*3*		*2344*	*7*	*21*
The investigator indicated the [target].	*hoof*	*124*	*1*	*3*		*4494*	*10*	*24*
The scientist looked at the [target].	*camel*	*358*	*2*	*5*		*17006*	*7*	*18*
The chairperson mentioned the [target]	*cannon*	*628*	*2*	*5*		*5236*	*7*	*21*
The president thought about the [target].	*bone*	*2348*	*1*	*3*		*21554*	*7*	*19*
The man remembered the [target].	*crocodile*	*247*	*3*	*8*		*76664*	*7*	*20*
The conductor presented the [target].	*ladder*	*1261*	*2*	*5*		*26538*	*8*	*23*
The guest pointed at the [target].	*paper*	*16755*	*2*	*5*		*19620*	*6*	*17*
The girl sketched the [target].	*arrow*	*1007*	*2*	*3*		*16138*	*4*	*12*
The father asked about the [target].	*pills*	*544*	*1*	*4*		*23319*	*6*	*17*
The reporter hid the [target].	*penguin*	*467*	*2*	*7*		*3955*	*6*	*18*
The artist moved towards the [target].	*bowl*	*2335*	*1*	*3*		*6774*	*6*	*20*
The secretary dreamed about the [target].	*swing*	*1880*	*1*	*4*		*21333*	*8*	*23*
The mother photographed the [target].	*sword*	*1330*	*1*	*4*		*27976*	*6*	*18*
The boy described the [target].	*sun*	*11025*	*1*	*3*		*27801*	*4*	*14*
The painter talked about the [target].	*table*	*19128*	*2*	*4*		*24251*	*7*	*18*
The queen painted the [target].	*volcano*	*378*	*3*	*7*		*11382*	*6*	*17*
The woman admired the [target].	*wool*	*1737*	*1*	*3*		*24271*	*6*	*14*
					AVG	23626	6	18
					SD	24361	1	4

Sentences Condition 3	Targets EN	Frequency	Syllables	Phonemes		Combined frequency indications of actor, verb & target	Combined syllable counts	Combined phoneme counts
The witness admired the [target].	*traffic light*	*37*	*3*	*9*		*3151*	*8*	*21*
The lady held the [target].	*beard*	*882*	*1*	*4*		*23098*	*4*	*12*

The king hid the [target].	*broom*	*323*	*1*	*4*		*18268*	*3*	*10*
The painter indicated the [target].	*pencil*	*1132*	*2*	*6*		*6315*	*8*	*21*
The secretary saw the [target].	*elephant*	*892*	*3*	*7*		*128887*	*8*	*18*
The father gazed at the [target].	*bottle*	*3900*	*2*	*4*		*26492*	*6*	*15*
The researcher moved towards the [target].	*fox*	*2245*	*1*	*3*		*3714*	*7*	*22*
The chairperson photographed the [target].	*dress*	*4739*	*1*	*4*		*7413*	*7*	*22*
The conductor observed the [target].	*pen*	*2072*	*1*	*3*		*4302*	*6*	*19*
The teacher remembered the [target].	*panda*	*175*	*2*	*5*		*27000*	*7*	*19*
The visitor presented the [target]	*wig*	*309*	*1*	*3*		*27077*	*7*	*20*
The queen talked about the [target].	*pirate*	*267*	*2*	*5*		*11789*	*6*	*17*
The boy pointed at the [target].	*radio*	*8454*	*3*	*5*		*21620*	*7*	*16*
The artist forgot about the [target].	*box*	*8363*	*1*	*3*		*12713*	*7*	*19*
The girl stood next to the [target].	*butterfly*	*623*	*3*	*8*		*15019*	*7*	*22*
The man sketched the [target].	*snail*	*288*	*1*	*4*		*59204*	*3*	*12*
The student dreamed about the [target].	*skeleton*	*627*	*3*	*8*		*12545*	*8*	*25*
The child drew the [target].	*skis*	*215*	*1*	*3*		*30791*	*3*	*10*
The reporter described the [target].	*mirror*	*3642*	*2*	*5*		*8995*	*7*	*21*
The guest asked about the [target].	*plug*	*876*	*1*	*4*		*3402*	*5*	*16*
The woman stared at the [target].	*rug*	*686*	*1*	*3*		*3360*	*5*	*16*
The investigator thought about the [target].	*clock*	*2764*	*1*	*4*		*6785*	*9*	*23*
The president looked at the [target].	*bird*	*3823*	*1*	*4*		*34036*	*6*	*19*
The mother painted the [target].	*curtain*	*1326*	*2*	*6*		*28859*	*6*	*17*
The scientist mentioned the [target].	*clothespin*	*654*	*2*	*8*		*7113*	*7*	*22*
					AVG	21278	6	18
					SD	26027	2	4

Sentences Practice trials	Targets EN	Frequency	Syllables	Phonemes		Combined frequency indications of actor, verb & target	Combined syllable counts	Combined phoneme counts
The clown sat next to the [target].	*drill*	*1033*	*1*	*3*		*1449*	*5*	*16*
The baby contemplated the [target].	*guitar*	*2401*	*2*	*5*		*11460*	*8*	*21*
The astronaut ran away from the [target].	*painting*	*4161*	*2*	*6*		*4384*	*9*	*25*
					AVG	5764	7	21
					SD	5146	2	5
