# Supplementary material for: Unpacking the multilingualism continuum: An investigation of language variety co-activation in simultaneous interpreters
Source: PLoS One. 2023 Nov 28;18(11):e0289484. doi: 10.1371/journal.pone.0289484 (PMC10684095; doi:10.1371/journal.pone.0289484)
Supplement: S3 Appendix — (PDF) [file pone.0289484.s003.pdf]

VISUAL WORLD STUDY, BIVARIETAL CO-ACTIVATION: MODELS USED FOR ANALYSIS

**GLMMs:**	binomial function ⇒ family = binomial, link = "logit"
**LMMs:**	REML = FALSE
**GCAs:**	control ⇒ lmerControl, optimizer = "bobyqa", REML = FALSE

COMPREHENSION TASK

GCA fixation proportion analysis (lmer* function*):

Full Model with all fixed effects considered:

Δ fixation proportions ([competitor fixations]-[distractor fixations]) ~ (ot1+ot2+ot3) * condition
+ task order + age + onset of fluency in German +
(1+(ot1+ot2+ot3)|Subject) +
(1+(ot1+ot2+ot3)|Subject:Condition)

Full Model with fixed effects relevant to the research question:

Δ fixation proportions ([competitor fixations]-[distractor fixations]) ~ (ot1+ot2+ot3) * condition
(1+(ot1+ot2+ot3)|Subject) +
(1+(ot1+ot2+ot3)|Subject:Condition)

Reduced Model (without interaction term; findings reported are from the reduced model where it provided the best fit as determined by the outcome of likelihood-ratio tests):

Δ fixation proportions ([competitor fixations]-[distractor fixations]) ~ (ot1+ot2+ot3) + condition +
(1+(ot1+ot2+ot3)|Subject) +
(1+(ot1+ot2+ot3)|Subject:Condition)

GLMM accuracy analysis (glmer* function*):

full model: Accuracy ~ Interpreter status * Bivarietalism status + Competitor type
+ (1|Subject) + (1|Item)

reduced model: Accuracy ~ Interpreter status + Bivarietalism status + Competitor type
+ (1|Subject) + (1|Item)

LMM RT analysis (lmer* function*):

full model: logRT ~ Interpreter status * Bivarietalism status + Competitor type
+ (1+Group|Subject) + (1+Group|Item)

reduced model: logRT ~ Interpreter status + Bivarietalism status + Competitor type
+ (1+Group|Subject) + (1+Group|Item)

PRODUCTION TASKS

GCA fixation proportion analysis (lmer* function*):

Full Model with all fixed effects considered:

Δ fixation proportions ([competitor fixations]-[distractor fixations]) ~ (ot1+ot2+ot3) * condition
+ task order + age + sentence rating/sentence strangeness + English onset +
(1+(ot1+ot2+ot3)|Subject) +
(1+(ot1+ot2+ot3)|Subject:Condition)

Full Model with fixed effects relevant to the research question:

Δ fixation proportions ([competitor fixations]-[distractor fixations]) ~ (ot1+ot2+ot3) * condition
(1+(ot1+ot2+ot3)|Subject) +
(1+(ot1+ot2+ot3)|Subject:Condition)

Reduced Model (without interaction term; findings reported are from the reduced models where it provided the best fit as determined by the outcome of likelihood-ratio tests):

Δ fixation proportions ([competitor fixations]-[distractor fixations]) ~ (ot1+ot2+ot3) + condition +
(1+(ot1+ot2+ot3)|Subject) +
(1+(ot1+ot2+ot3)|Subject:Condition)

GLMM accuracy analysis (glmer* function*):

full model: Accuracy ~ Bivarietalism status * Competitor type + (1|Subject) + (1|Item)
reduced model: Accuracy ~ Bivarietalism status + Competitor type + (1|Subject) + (1|Item)

LMM RT analysis (lmer* function*):

full model: logRT ~ Bivarietalism status * Competitor type + (1+Group|Subject) + (1+Group|Item)
reduced model: logRT ~ Bivarietalism status + Competitor type + (1+Group|Subject) + (1+Group|Item)
