# Supplementary material for: Unpacking the multilingualism continuum: An investigation of language variety co-activation in simultaneous interpreters
Source: PLoS One. 2023 Nov 28;18(11):e0289484. doi: 10.1371/journal.pone.0289484 (PMC10684095; doi:10.1371/journal.pone.0289484)
Supplement: S4 Appendix — (PDF) [file pone.0289484.s004.pdf]

**Models scripts and outputs of the Growth Curve Analyses conducted**

R scripts created by Dr. Malte C. Viebahn

*Data preparation prior to analysis:*

- *Create data subsets by condition*
- *Analogous approach used to create subsets and plots by group, interpreter status and bivarietalism status as well as task type*
- *"meanFix" = average number of fixations per time bin per condition per participant*
- *"sumFix" = the number of trials on which the target was fixated by each participant in each condition in each time bin*

***Comprehension task data analysis script for illustration purposes:***

```
MakePPDatasetByConfl <- function(mydata) { # create subset per group
   meanFix.dat <- with(mydata, aggregate(list(t.meanFix=fixcount.target,
                                   c.meanFix=fixcount.comp,
                                   d1.meanFix=fixcount.distr1,
                                   d2.meanFix=fixcount.distr2,
                                   d3.meanFix=fixcount.distr3,
                                   o.meanFix=fixcount.other),
                                      list(time=bin, pp=pp.id, condition=condition,
                                      defluency=defluency),mean))

   N.dat <- with(mydata, aggregate(list(N.trials=fixcount.total),
                 list(time=bin, pp=pp.id, condition=condition, defluency=defluency), length))

   sumFix.dat <- with(mydata, aggregate(list(t.sumFix=fixcount.target, c.sumFix=fixcount.comp,
   d1.sumFix=fixcount.distr1, d2.sumFix=fixcount.distr2, o.sumFix=fixcount.other),
                              list(time=bin, pp=pp.id, condition=condition, defluency=defluency),
                              sum))

   meanFix.dat$d.meanFix.mean  <- with(meanFix.dat, (d1.meanFix + d2.meanFix)/2)
   meanFix.dat$tc.meanFix.diff <- with(meanFix.dat, t.meanFix - c.meanFix)
   meanFix.dat$cd.meanFix.diff <- with(meanFix.dat, c.meanFix - d.meanFix.mean)
   meanFix.dat$condy           <- with(meanFix.dat, ifelse(condition=="No competitor",
         (d1.meanFix+d2.meanFix+d3.meanFix)/3, c.meanFix))

   combined.dat <- cbind(meanFix.dat, sumFix.dat[,4:8])
   combined.dat$N.trials <- N.dat[,4]

   combined.dat$t.elog <- with(combined.dat, CalcElogit(Y=t.sumFix, N=N.trials))
   combined.dat$t.wts  <- with(combined.dat, CalcWeight(Y=t.sumFix, N=N.trials))

   combined.dat$c.elog <- with(combined.dat, CalcElogit(Y=c.sumFix, N=N.trials))
   combined.dat$c.wts  <- with(combined.dat, CalcWeight(Y=c.sumFix, N=N.trials))

combined.dat$tc.elog <- with(combined.dat, CalcElogit(Y=t.sumFix, N=(t.sumFix + c.sumFix)))
   combined.dat$tc.wts  <- with(combined.dat, CalcWeight(Y=t.sumFix, N=(t.sumFix + c.sumFix)))
```

```
    #combined.dat$cd.elog <- with(combined.dat, CalcElogit(Y=c.sumFix, N=(c.sumFix + d1.sumFix
    + d2.sumFix)))
    #combined.dat$cd.wts  <- with(combined.dat, CalcWeight(Y=c.sumFix, N=(c.sumFix + d1.sumFix
    + d2.sumFix)))

    combined.dat$cd.elog <- with(combined.dat, CalcElogit(Y=c.sumFix, N=(c.sumFix + ((d1.sumFix
    + d2.sumFix)/2))))
    combined.dat$cd.wts  <- with(combined.dat, CalcWeight(Y=c.sumFix, N=(c.sumFix +
    ((d1.sumFix + d2.sumFix)/2))))

    return(combined.dat)
}


MakeGCADatasetCondition <- function(mydata, bin.min, bin.size, n.poly){
  mydata$bin.num <- (mydata$time - bin.min + bin.size)/bin.size
  t <- poly(unique(mydata$bin.num), n.poly)
  mydata[, paste('ot', 1:n.poly, sep='')] <- t[mydata$bin.num, 1:n.poly]
  mydata$condition <- as.factor(as.character(mydata$condition))
  return(mydata)
}


ModelPlot for Condition

c.meanFix.ModelPlotCondition <- function(mymodel, mytitle) {
  mydata <- get(as.character(mymodel@call$data))
  ggplot(mydata, aes(time, condy, color=condition)) +
    coord_cartesian(ylim=c(0, .25)) +
    scale_colour_grey() +
    theme_bw() +
    #scale_y_continuous(breaks=seq(from=.1, to=.75, by=.1)) +
    scale_y_continuous(breaks=seq(from=0, to=.25, by=.05)) +
    stat_summary(fun.data=mean_se, geom="pointrange") +
    labs(title=mytitle, y="Fixation proportion per condition", x="Time from target word onset (10 ms
time bins)") +
    guides(linetype=guide_legend(ncol=1)) +
    #theme_linedraw() +
    #theme_bw() +
    theme(legend.position='top',
          legend.text=element_text(size=10),
          legend.title=element_blank(),
          plot.title=element_text(size=10),
          axis.title=element_text(size=10),
          axis.text.x=element_text(size=10, colour='black'),
          axis.text.y=element_text(size=10, colour='black'))
}
```

```
fx.all.df                     <- allfixcount.df
fx.all.pp.df                  <- MakePPDatasetByCon(fx.all.df)
fx.all.pp.400.1000.df         <- MakeGCADatasetGroup(mydata=subset(fx.all.pp.df, time>=400 &
                                    time<=1000), bin.min=400, bin.size=10, n.poly=3)
fx.all.pp.400.1000.df$group = as.factor(fx.all.pp.400.1000.df$group)
fx.all.pp.400.1000.df$group   <- relevel(fx.all.pp.400.1000.df$group, ref='Diglossics.Non-interpreters')
```

```
fx.all.pp.400.1000.lmer1        <- lmer(justcomp ~ (ot1+ot2+ot3) * group + age + cf + enprof +
                                 (1+(ot1+ot2+ot3)|pp) +
                                 (1+(ot1+ot2+ot3)|pp:condition),
                                 control=lmerControl(optimizer="bobyqa", optCtrl=list(maxfun=20000)),
                                 REML=F, data=fx.all.pp.400.1000.df)

summary(fx.all.pp.400.1000.lmer1)
```

*Plot function for the GCA plots provided for the paper:*

```
fx.all.pp.400.1000.gca.plot     <- c.meanFix.ModelPlotJustComp(mymodel=fx.all.pp.400.1000.lmer1,
                                 mytitle='All groups just comp')
fx.all.pp.400.1000.gca.plot + stat_summary(aes(y=fitted(fx.all.pp.400.1000.lmer1), color=group),
fun.y=mean, geom="line")
```

*Model output (→summary) for fixed effects comparing the different groups' same-variety activation levels with the performance of bivarietal (biv.) non-interpreters as baseline (monov = monovarietal):*

| | Estimate | Std. Error | df | t value |
|---|---|---|---|---|
| (Intercept) | 0.390946 | 0.038633 | 64.000000 | 10.119 *** |
| ot1 (linear time term) | 1.527596 | 0.164693 | 64.040000 | 9.275 *** |
| ot2 (quadratic time term) | -0.019342 | 0.069902 | 64.010000 | -0.277 |
| ot3 (cubic time term) | -0.154108 | 0.042342 | 64.010000 | -3.640 *** |
| groupBiv.Interpreters | 0.025704 | 0.054636 | 64.000000 | 0.470 |
| groupMonov.Interpreters | 0.055093 | 0.054636 | 64.000000 | 1.008 |
| group Monov.Non-interpreters | 0.039864 | 0.054636 | 64.000000 | 0.730 |

*Model output for fixed effects comparing the two bivarietal groups' cross-variety activation levels:*

| | Estimate | Std. Error | df | t value |
|---|---|---|---|---|
| (Intercept) | 0.0797541 | 0.0102863 | 32.0100000 | 7.753 *** |
| ot1 | -0.2524295 | 0.0561368 | 32.0200000 | -4.497 *** |
| ot2 | -0.0673271 | 0.0427184 | 32.0300000 | -1.576 |
| ot3 | 0.0585815 | 0.0285196 | 32.0100000 | 2.054 * |
| groupBiv.Interpreters | -0.0007838 | 0.0145470 | 32.0100000 | -0.054 |
| ot1:groupBiv.Interpreters | -0.1363547 | 0.0793895 | 32.0200000 | -1.718 |
| ot2:groupBiv.Interpreters | 0.0781452 | 0.0604129 | 32.0300000 | 1.294 |
| ot3:groupBiv.Interpreters | -0.0184496 | 0.0403328 | 32.0100000 | -0.457 |

*Model output for fixed effects comparing the two bivarietal groups' same-variety activation levels:*

| | Estimate | Std. Error | df | t value | |
|---|---|---|---|---|---|
| (Intercept) | 0.08522 | 0.01173 | 31.99000 | 7.264 *** | |
| ot1 | -0.21785 | 0.06789 | 31.99000 | -3.209 ** | |
| ot2 | -0.01392 | 0.04069 | 32.00000 | -0.342 | |
| ot3 | 0.02786 | 0.02567 | 32.00000 | 1.086 | |
| groupDiglossics.Interpreters | 0.02063 | 0.01659 | 31.99000 | 1.243 | |
| ot1:groupDiglossics.Interpreters | -0.21388 | 0.09601 | 31.99000 | -2.228 * | |
| ot2:groupDiglossics.Interpreters | -0.01424 | 0.05754 | 32.00000 | -0.247 | |
| ot3:groupDiglossics.Interpreters | 0.01929 | 0.03630 | 32.00000 | 0.531 | 0.59889 |

*Model output for fixed effects comparing the two interpreter groups' same-variety activation levels:*

| | Estimate | Std. Error | df | t value |
|---|---|---|---|---|
| (Intercept) | 0.105847 | 0.010203 | 32.010000 | 10.374 *** |
| ot1 | -0.431734 | 0.062286 | 32.020000 | -6.931 *** |
| ot2 | -0.028157 | 0.042636 | 32.000000 | -0.660 |
| ot3 | 0.047151 | 0.026732 | 32.000000 | 1.764 |
| groupNon-diglossics.Interpreters | -0.005819 | 0.014429 | 32.010000 | -0.403 |
| ot1:groupNon-diglossics.Interpreters | -0.013106 | 0.088086 | 32.020000 | -0.149 |
| ot2:groupNon-diglossics.Interpreters | 0.020435 | 0.060296 | 32.000000 | 0.339 |
| ot3:groupNon-diglossics.Interpreters | 0.052391 | 0.037805 | 32.000000 | 1.386 |

*Model output for between group comparisons to check for between-group differences in fixation proportions*

| | Estimate | Std. Error | t value |
|---|---|---|---|
| (Intercept) | 5.408e-02 | 9.453e-03 | 5.721 * |
| ot1 | -2.889e-01 | 5.242e-02 | -5.512 * |
| ot2 | 5.693e-02 | 3.300e-02 | 1.725 |
| ot3 | 1.745e-02 | 2.349e-02 | 0.743 |
| conditionStd. Ger. comp. | 5.177e-02 | 1.005e-02 | 5.150 * |
| conditionSwiss Ger. comp. | 2.489e-02 | 1.005e-02 | 2.476 * |
| groupDiglossics.Non-interpreters | 1.833e-03 | 1.337e-02 | 0.137 |
| groupNon-diglossics.Interpreters | 5.090e-03 | 1.337e-02 | 0.381 |
| groupNon-diglossics.Non-interpreters | -4.616e-04 | 1.337e-02 | -0.035 |
| ot1:conditionStd. Ger. comp. | -1.428e-01 | 6.244e-02 | -2.287 * |
| ot1:conditionSwiss Ger. comp. | -9.987e-02 | 6.244e-02 | -1.599 |
| ot2:conditionStd. Ger. comp. | -8.509e-02 | 4.346e-02 | -1.958 |
| ot2:conditionSwiss Ger. comp. | -4.611e-02 | 4.346e-02 | -1.061 |
| ot3:conditionStd. Ger. comp. | 2.970e-02 | 3.048e-02 | 0.974 |
| ot3:conditionSwiss Ger. comp. | 2.268e-02 | 3.048e-02 | 0.744 |
| ot1:groupDiglossics.Non-interpreters | 5.943e-02 | 7.413e-02 | 0.802 |
| ot1:groupNon-diglossics.Interpreters | 2.907e-02 | 7.413e-02 | 0.392 |
| ot1:groupNon-diglossics.Non-interpreters | 7.475e-02 | 7.413e-02 | 1.008 |
| ot2:groupDiglossics.Non-interpreters | -1.719e-02 | 4.667e-02 | -0.368 |
| ot2:groupNon-diglossics.Interpreters | -3.662e-03 | 4.667e-02 | -0.078 |
| ot2:groupNon-diglossics.Non-interpreters | -5.043e-03 | 4.667e-02 | -0.108 |
| ot3:groupDiglossics.Non-interpreters | 8.176e-05 | 3.322e-02 | 0.002 |
| ot3:groupNon-diglossics.Interpreters | -2.696e-02 | 3.322e-02 | -0.812 |
| ot3:groupNon-diglossics.Non-interpreters | 4.495e-03 | 3.322e-02 | 0.135 |
| conditionStd. Ger. comp.:groupDiglossics.Non-interpreters | -2.246e-02 | 1.421e-02 | -1.580 |
| conditionSwiss Ger. comp.:groupDiglossics.Non-interpreters | -1.050e-03 | 1.421e-02 | -0.074 |
| conditionStd. Ger. comp.:groupNon-diglossics.Interpreters | -1.091e-02 | 1.421e-02 | -0.767 |
| conditionSwiss Ger. comp.:groupNon-diglossics.Interpreters | -2.287e-02 | 1.421e-02 | -1.609 |
| conditionStd. Ger. comp.:groupNon-diglossics.Non-interpreters | -1.944e-02 | 1.421e-02 | -1.368 |
| conditionSwiss Ger. comp.:groupNon-diglossics.Non-interpreters | -2.466e-02 | 1.421e-02 | -1.735 |
| ot1:conditionStd. Ger. comp.:groupDiglossics.Non-interpreters | 1.545e-01 | 8.831e-02 | 1.749 |
| ot1:conditionSwiss Ger. comp.:groupDiglossics.Non-interpreters | 7.693e-02 | 8.831e-02 | 0.871 |
| ot1:conditionStd. Ger. comp.:groupNon-diglossics.Interpreters | -4.218e-02 | 8.831e-02 | -0.478 |
| ot1:conditionSwiss Ger. comp.:groupNon-diglossics.Interpreters | 1.260e-01 | 8.831e-02 | 1.427 |
| ot1:conditionStd. Ger. comp.:groupNon-diglossics.Non-interpreters | 4.449e-02 | 8.831e-02 | 0.504 |
| ot1:conditionSwiss Ger. comp.:groupNon-diglossics.Non-interpreters | 4.985e-02 | 8.831e-02 | 0.565 |
| ot2:conditionStd. Ger. comp.:groupDiglossics.Non-interpreters | 3.143e-02 | 6.146e-02 | 0.511 |
| ot2:conditionSwiss Ger. comp.:groupDiglossics.Non-interpreters | -6.095e-02 | 6.146e-02 | -0.992 |
| ot2:conditionStd. Ger. comp.:groupNon-diglossics.Interpreters | 2.410e-02 | 6.146e-02 | 0.392 |

| | | | |
|---|---|---|---|
| ot2:conditionSwiss Ger. comp.:groupNon-diglossics.Interpreters | -1.150e-02 | 6.146e-02 | -0.187 |
| ot2:conditionStd. Ger. comp.:groupNon-diglossics.Non-interpreters | -1.257e-02 | 6.146e-02 | -0.205 |
| ot2:conditionSwiss Ger. comp.:groupNon-diglossics.Non-interpreters | 4.861e-02 | 6.146e-02 | 0.791 |
| ot3:conditionStd. Ger. comp.:groupDiglossics.Non-interpreters | -1.937e-02 | 4.311e-02 | -0.449 |
| ot3:conditionSwiss Ger. comp.:groupDiglossics.Non-interpreters | 1.837e-02 | 4.311e-02 | 0.426 |
| ot3:conditionStd. Ger. comp.:groupNon-diglossics.Interpreters | 7.935e-02 | 4.311e-02 | 1.841 |
| ot3:conditionSwiss Ger. comp.:groupNon-diglossics.Interpreters | -2.378e-03 | 4.311e-02 | -0.055 |
| ot3:conditionStd. Ger. comp.:groupNon-diglossics.Non-interpreters | 9.356e-03 | 4.311e-02 | 0.217 |
| ot3:conditionSwiss Ger. comp.:groupNon-diglossics.Non-interpreters | -1.773e-02 | 4.311e-02 | -0.411 |

*The model output provided above are from the analyses run on the comprehension task data. For illustration purposes, the model output for between group comparisons for the production task performed by the interpreter groups is provided below. As can be gathered from the output, the data does not indicate a phonological competitor effect for either of the interpreters' target language varieties (a finding repeated for all the analyses we ran):*

| | Estimate | Std. Error | df | t value |
|---|---|---|---|---|
| (Intercept) | 0.078543 | 0.015147 | 21.410000 | 5.185 *** |
| ot1 | -0.145799 | 0.062789 | 40.500000 | -2.322 * |
| ot2 | -0.034288 | 0.046705 | 47.830000 | -0.734 |
| ot3 | 0.006508 | 0.022295 | 46.640000 | 0.292 |
| conditionStd. Ger. comp. | 0.014627 | 0.009861 | 32.040000 | 1.483 |
| conditionSwiss Ger. comp. | 0.013608 | 0.009861 | 32.040000 | 1.380 |
| ot1:conditionStd. Ger. comp. | 0.088124 | 0.073991 | 33.460000 | 1.191 |
| ot1:conditionSwiss Ger. comp. | -0.007952 | 0.073991 | 33.460000 | -0.107 |
| ot2:conditionStd. Ger. comp. | -0.001223 | 0.064573 | 46.260000 | -0.019 |
| ot2:conditionSwiss Ger. comp. | 0.022304 | 0.064573 | 46.260000 | 0.345 |
| ot3:conditionStd. Ger. comp. | -0.004578 | 0.029563 | 39.370000 | -0.155 |
| ot3:conditionSwiss Ger. comp. | -0.005936 | 0.029563 | 39.370000 | -0.201 |
