# Supplementary material for: Unpacking the multilingualism continuum: An investigation of language variety co-activation in simultaneous interpreters
Source: PLoS One. 2023 Nov 28;18(11):e0289484. doi: 10.1371/journal.pone.0289484 (PMC10684095; doi:10.1371/journal.pone.0289484)
Supplement: S5 Appendix — (PDF) [file pone.0289484.s005.pdf]

UNIVERSITÉ DE GENÈVE
FACULTÉ DE TRADUCTION ET D'INTERPRÉTATION

INFORMED CONSENT

Dear participant,

Thank you for your willingness to participate in the research project described below. It is part of Laura Keller's PhD project supervised by Prof. K. Seeber, University of Geneva and Prof. L. Roberts, University of York.

Description of the project:

This experiment is part of the study *Language processing in the multilingual mind: Exploring target language selection in simultaneous interpreting*, attempting to test the mechanisms allowing for targeted production language selection in a high proficiency multilingual setting involving simultaneous comprehension and production. It was approved by the FTI's Ethics Committee and the experiment takes about 75 minutes to complete. Should you have further questions Laura Keller will discuss them with you in detail.

Risks or discomfort:

The experimental procedure is non-invasive and involves no health risk or hazard. While you carry out some of the experimental tasks, your pupils will be recorded with an infrared camera. The measures taken are indicatory of language processing but cannot be influenced arbitrarily.

Confidentiality:

Your participation in this study is anonymous and confidential. The FTI will protect your privacy and the confidentiality of the data gathered in the course of this experiment within legal limits. All records of your performance will be stored anonymously and destroyed after five years.

Voluntary participation and withdrawal:

Participation in this research is voluntary. You have the right to refuse to be in this study. If you decide to be in the study and change your mind, you have the right to drop out at any time.

Questions, rights and complaints:

If you have any questions about this research project, please contact LaborInt@unige.ch or a member of the Faculty's Ethics Committee.

Results:

If you would like to be informed about the results of the study, please indicate your email address below.

Consent statement:

By signing this document, I consent to participating in this experiment under the aforementioned conditions. I certify that I am 18 years of age and that I have read and understood the consent form.

____
Signature of Participant

____
Typed/printed Name

____
Date

____
Email of Participant

____
Signature of researcher
