# Supplementary material for: Unpacking the multilingualism continuum: An investigation of language variety co-activation in simultaneous interpreters
Source: PLoS One. 2023 Nov 28;18(11):e0289484. doi: 10.1371/journal.pone.0289484 (PMC10684095; doi:10.1371/journal.pone.0289484)
Supplement: Attachment — Submitted filename: Response to Reviewers.pdf [file pone.0289484.s008.pdf]

Laura Keller, Ph.D.
Faculty of Translation and Interpreting
University of Geneva
Avenue du Pont-d'Arve 40
CH-1205 Geneva

Nicola Molinaro, Ph.D.
Academic Editor
PLOS ONE

Geneva, 14th July 2023

Dear Dr. Molinaro,

Many thanks for the careful consideration our responses to the first round of reviews were given and for the quick turn-around. We would like to hereby submit a revised version of our manuscript in which we addressed the additional comments made by the Reviewer 2.

As for our previous resubmission, we take up the individual requests printed in *blue* and elaborate on how we addressed the suggestions made in detail below each request in black.

FEEDBACK REVIEWER 1

No additional comments made.

FEEDBACK REVIEWER 2

I am very happy to see that the authors have done an excellent job at addressing my comments.

We would like to thank Reviewer 2 for their feedback on our submission following the first round of reviews, we really appreciate it.

I only have a few minor comments at this point.

1. *In the previous round of review, I had asked the authors to clarify their interpretation of the cubic term parameter (i.e., the cognitive process reflected in that parameter). The authors kindly responded that they interpreted the significant effect of this factor "as an indicator of lower fixation proportions on cross-variety competitors in the bivarietal interpreter group, as it pointed to a shallower curvature". I thank the authors for adding this; however, it remains unclear to me how exactly this maps onto the underlying cognitive processes - that is, my question was referring to the linking hypothesis (see Allopenna et al., 1998). For example, in static analytical approaches (e.g., average fixation probability) the extracted measure is thought to reflect overall degree of lexical activation for that item, whereas in a dynamic approach such as curvefitting (Seedorff et al, 2018), slope can be interpreted as the speed at which lexical activation builds up. Based on the authors' response, my understanding is that they take the cubic term parameter to reflect the overall amount of lexical activation; however, in that case, I am not sure how this effect differs from an intercept effect (this is why I voiced my question in the previous round as to the advantage of using a dynamic analytical approach such as GCA instead of a traditional fixation probability measure). In any case, I don't think this is highly problematic, but I think that the paper would benefit from a clearer description of what the cubic term is thought to reflect –not just in terms of fixations, but rather in terms of the underlying cognitive processes.*

Thank you for clarifying your question and our apologies for not getting to the intended point with our first attempt to address it. Indeed, the cubic term parameter was taken as an indicator of the curvature of lexical

activation as expressed in visual attention. We based this on the assumption that lexical activation is not a process that can be captured in a linear manner, but that the incremental nature of language processing (and according to our hypothesis therefore also of language and language variety co-activation) would follow a sinusoidal rather than a linear or sigmoidal curvature, indicating an increase and subsequent decrease of visual attention after the disambiguation point during comprehension, rather than a continuous shift (Guedes et al., 2022). Although GCA is far from being the only valid approach to analysing visual world fixation data (see e.g. Seedorff et al., 2018), we opted for this analytical approach in an attempt to capture the up-and-down movement expected in terms of fixation proportions to the phonological competitor in a time window selected according to a theoretical rationale ahead of the analysis, based on the time estimated necessary according to evidence from the literature for lexical access and planning and executing the eye movement (Seedorf et al., 2018, point out in particular that high order polynomials did not live up to the promise of capturing these types of fluctuations well, however, we would argue that a third order polynomial not that high and the GCA still allows to jointly account for by-subject and by-item variability). We included this rationale in the manuscript (lines 673-689 in *Revised Manuscript with Track Changes*).

2. *At the beginning of the "Fixation data" section, it is mentioned that "The fixation-time course observed – a divergence of visual attention starting at around 400 ms after target-word onset – corresponds to the 200 ms estimated for lexical access [78] added to 200 ms necessary to plan an eye movement [79]". I just wanted to point out that this is perhaps a bit late, given that the typical timing of such effects is ~200-300 ms (see Allopenna et al., 1998). I'm only mentioning this, because the wording right now makes it sound as if 400 ms is the earliest this type of effect can show up, which is clearly not the case.*

Indeed, the idea was to argue that the 400ms could be explained by adding the time spans reported in the literature for the execution of the two processes (lexical access and eye movement planning), not that this was the earliest and only moment at which such an effect could be observed in the data. We rephrased the sentence in question to introduce that missing nuance (lines 454-457).

3. *In response to a previous question, the authors added that "RTs for the production task (trial onset to target-word onset in the participants' production) were extracted from the audio files using a Praat script". If this script is publically available, it would be helpful to add a link so that that an interested reader can see the details of the extraction procedure. Also, were these data checked by an experimenter?*

The Praat script we refer to here was written by MCV and is not publicly available. However, during the verification process of the output produced, which was the basis to determine the accuracy scores, the output generated by the Praat script was checked by the experimenter (LK). The manuscript was updated to reflect the post-Praat verification step (lines 565-566).

4. *There's a typo at line 677: "more efficiently then the bivarietal non-interpreters" --> "more efficiently than the bivarietal non-interpreters"*

Thank you for the care taken in reading the manuscript again, we corrected the typo in the manuscript (line 702).

As mentioned above, these are all minor comments. Overall, I'm very happy with how the authors addressed previous comments and I'm looking forward to seeing this work published soon.

Again, a heartfelt thank you for taking the time to go through our attempts to address your comments so diligently, and for making the effort of including constructive criticism as well as praise in your feedback. We also really appreciated the additional references (Allopenna et al., 1998; Seedorf et al., 2018) that we were happy to include in our manuscript.

We would like to thank Reviewer 2 for going over our resubmitted manuscript one more time with such great attention and for again taking the time to address pertinent comments to help us further improve our paper. This degree of effort is exceptional, and we are grateful for the time invested in reviewing our work.

My co-authors, Dr. Malte Viebahn, Prof. Dr. Alexis Hervais-Adelman, Prof. Dr. Kilian Seeber, and I hope that the additional changes made to the manuscript will allow you to consider it for publication in PLOS ONE.

Sincerely,

Laura Keller, Ph.D.
